# A Qualitative Exploration of the Acceptability of an Online Web Application to Promote Breast and Cervical Cancer Screening in Primary Care Settings in Malaysia

**DOI:** 10.1200/GO.22.48000

**Published:** 2022-05-05

**Authors:** Ai Theng Cheong, Ping Yien Lee, Sazlina Shariff Ghazali, Aneesa Abdul Rashid, Chirk Jenn Ng, Chin Hai Teo, Yamuna Rajoo, Ong Siu Ching, Maheswari Jaganathan, Bee Kiau Ho, Soo-Hwang Teo

**Affiliations:** ^1^ Department of Family Medicine, Universiti Putra Malaysia, Seri Kembangan, Malaysia; ^2^ Department of Primary Care Medicine, University of Malaya, Kuala Lumpur, Malaysia; ^3^ Cancer Research Malaysia, Subang Jaya, Malaysia; ^4^ Klinik Kesihatan Bandar Botanik, Port Klang, Malaysia; ^5^ Cancer Research Malaysia; University Malaya Cancer Research Institute, Kuala Lumpur, Malaysia

## Abstract

In Malaysia, breast and cervical are the most common and third most common cancers respectively. They are associated with high morbidity and mortality in women. With the rapid development of information communication technology and widespread adoption of mobile devices in low to middle-income countries, online web applications (app) could be one of the strategies to engage patients to go for screening programs.

**METHODS:**

Fifteen (15) health care professionals (HCPs) and 25 patients visiting the primary care were interviewed through focus groups and in-depth interviews to determine the acceptability of an online web application to promote screening for these cancers.

**RESULTS:**

Both HCPs and patients expressed that an online web app would be beneficial to the technology-savvy group such as the younger generation. However, the older-aged group could benefit from it with some assistance. The primary consideration identified is the user's information technology (IT) competency. Participants suggested that the use of easy-to-use features (eg, download method), visually easy-to-understand formats (eg, less text and more pictures, videos), and provision of support to navigate the web app may be critical to ensure good uptake of a web app on screening. Contents of the app suggested to be included were: individual risk assessment for the users of the app, benefits of screening, information addressing patients' barriers such as fear, embarrassment, logistic, and cost issues.

**CONCLUSION:**

The findings of our study may be useful as a guide to developing an online web app to promote breast and cervical cancer screening.

